# The genome sequence of Langmaid’s Yellow Underwing moth, *Noctua janthina *(Denis & Schiffermüller) 1775

**DOI:** 10.12688/wellcomeopenres.23192.1

**Published:** 2024-10-15

**Authors:** Gavin R. Broad, Natural History Museum Genome Acquisition Lab, Darwin Tree of Life Barcoding collective, Wellcome Sanger Institute Tree of Life Management, Samples and Laboratory team, Wellcome Sanger Institute Scientific Operations: Sequencing Operations, Wellcome Sanger Institute Tree of Life Core Informatics team, Tree of Life Core Informatics collective, Darwin Tree of Life Consortium

**Affiliations:** 1 Natural History Museum, London, England, UK

**Keywords:** Noctua janthina, Langmaid’s Yellow Underwing moth, genome sequence, chromosomal, Lepidoptera

## Abstract

We present a genome assembly from an individual male *Noctua janthina* (Langmaid’s Yellow Underwing; Arthropoda; Insecta; Lepidoptera; Noctuidae). The genome sequence has a total length of 539.70 megabases. Most of the assembly (99.99%) is scaffolded into 31 chromosomal pseudomolecules, including the Z sex chromosome. The mitochondrial genome has also been assembled and is 15.36 kilobases in length. Gene annotation of this assembly on Ensembl identified 12,089 protein-coding genes.

## Species taxonomy

Eukaryota; Opisthokonta; Metazoa; Eumetazoa; Bilateria; Protostomia; Ecdysozoa; Panarthropoda; Arthropoda; Mandibulata; Pancrustacea; Hexapoda; Insecta; Dicondylia; Pterygota; Neoptera; Endopterygota; Amphiesmenoptera; Lepidoptera; Glossata; Neolepidoptera; Heteroneura; Ditrysia; Obtectomera; Noctuoidea; Noctuidae; Noctuinae; Noctuini; *Noctua*; *Noctua janthina* (Denis & Schiffermüller) 1775 (NCBI:txid987996).

## Background

*Noctua janthina*, Langmaid’s Yellow Underwing, is one of three very similar species until relatively recently confused under the name ‘Lesser Broad-bordered Yellow Underwing’. All three are relatively small *Noctua* moths with a limited bright yellow spot on the hind wing and a fore wing which is basically dark brown with variable subtle purple and red tints. Before Mentzner *et al.* (1991), the name *Noctua janthina* (Denis & Schiffermüller) was used in all the standard guides for the traditional ‘Lesser Broad-bordered Yellow Underwing’ (e.g., Skinner, 1984), but the species which is widespread and common in northern Europe is now recognized as *Noctua janthe* Borkhausen ( Mentzner *et al.*, 1991). The true *Noctua janthina*, which we have sequenced here, was first recorded from southern England by Langmaid (2002), and was then given the vernacular, ‘Langmaid’s Yellow Underwing’. This species seems to be established around the South coast of England, particularly in Kent, with numbers boosted by immigration ( Clancy *et al.*, 2012; Waring *et al.*, 2017). Recently, the third species in the complex, *Noctua tertia* Mentzner, Moberg & Fibiger, has been found in the UK ( Elliott & Wilson, 2022).

Identification of *Noctua janthina* can be tricky. Potential specimens can often be singled out by their slightly duller colour than *N. janthe* (although colour pattern is very variable) and particularly by the shorter wings. The hind wing pattern is more reliable, with the yellow patch virtually surrounded by black borders, whereas the black border is narrower on *N. janthe*, with a large gap on the leading edge, although *N. tertia* is very similar to *N. janthina*. The rather minor but consistent differences in genitalia are illustrated by Mentzner *et al.* (1991) and by Beck (2013).

Larvae don’t seem to have been found in the UK ( Waring *et al.*, 2017), but are illustrated and diagnosed by Beck (2013). The life history is expected to resemble that of *N. janthe*. Adults are active from June to September, particularly in July and August (e.g., Perry, no date), a little earlier than the peak for *N. janthe*. Larvae presumably feed on a wide range of herbaceous plants from autumn through to spring, pupating in the soil in late spring. Adults are readily attracted to light.

Following the release of the genomes of *Noctua fimbriata* ( Holland *et al.*, 2021), *N. pronuba* ( Boyes *et al.*, 2022), *N. janthe* ( Boyes *et al.*, 2023) and *N. comes* ( Boyes *et al.*, 2024), this genome adds to a growing number of Noctuinae genomes, including those of closely related species, and will help in understanding the huge success of this group of moths.

## Genome sequence report

**Figure 1.**
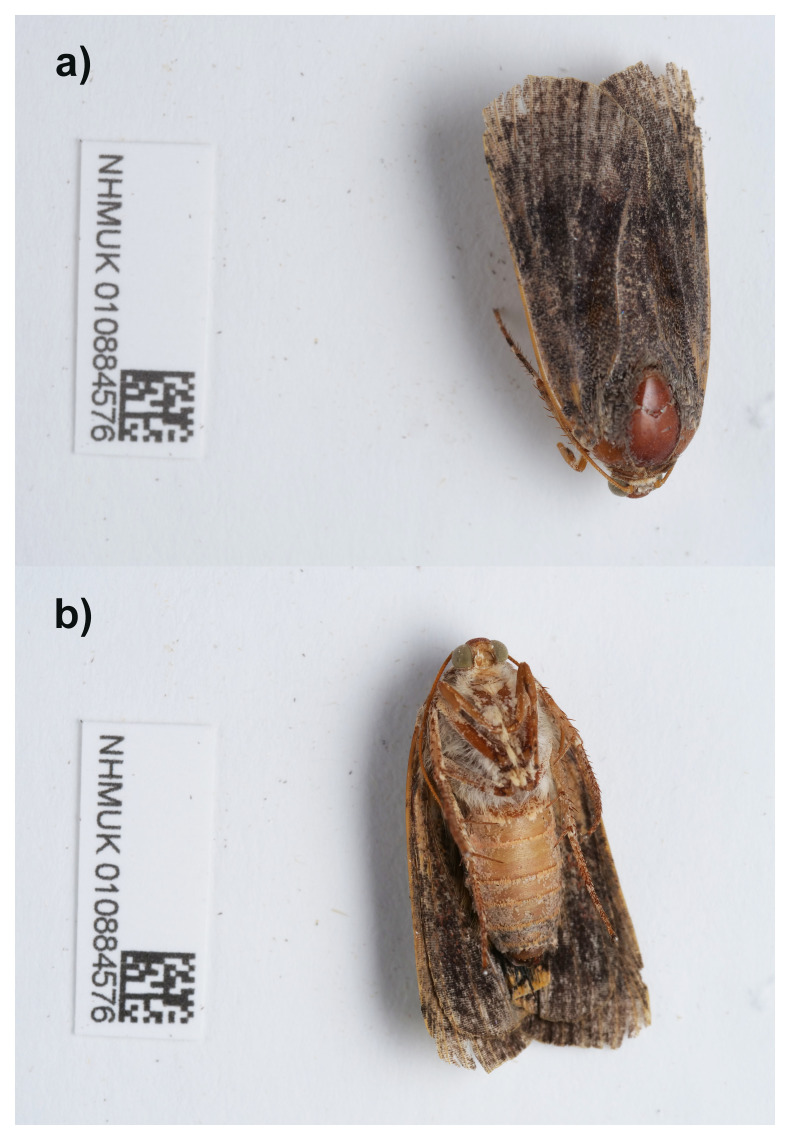
Photographs of the *Noctua janthina* (ilNocJanh1) specimen used for genome sequencing. **a**) Dorsal view, **b**) Ventral view.

The genome of an adult male *Noctua janthina* ( Figure 1) was sequenced using Pacific Biosciences single-molecule HiFi long reads, generating a total of 42.15 Gb (gigabases) from 3.95 million reads, providing approximately 75-fold coverage. Primary assembly contigs were scaffolded with chromosome conformation Hi-C data, which produced 133.15 Gb from 881.79 million reads, yielding an approximate coverage of 247-fold. Specimen and sequencing information is summarised in Table 1.

**Table 1.** Specimen and sequencing data for *Noctua janthina*.

Project information			
**Study title**	*Noctua janthina*		
**Umbrella BioProject**	PRJEB67419		
**BioSample**	SAMEA112964405		
**NCBI taxonomy ID**	987996		
Specimen information			
**Technology**	**ToLID**	**BioSample accession**	**Organism part**
**PacBio long read sequencing**	ilNocJanh1	SAMEA112975574	Whole organism
**Hi-C sequencing**	ilNocJanh1	SAMEA112975574	Whole organism
Sequencing information			
**Platform**	**Run accession**	**Read count**	**Base count (Gb)**
**Hi-C Illumina NovaSeq 6000**	ERR12121870	8.82e+08	133.15
**PacBio Revio**	ERR12120046	3.95e+06	42.15

**Figure 2.**
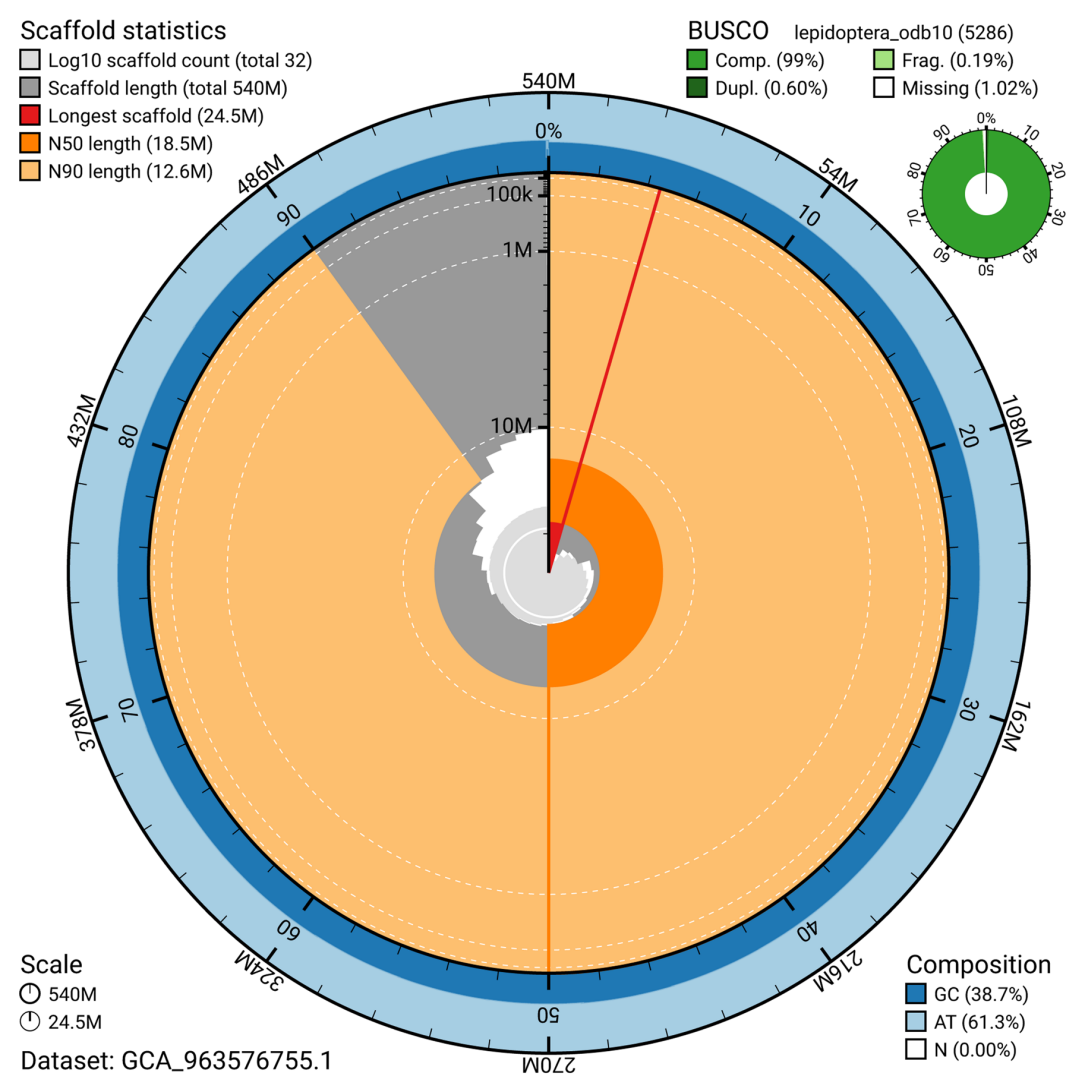
Genome assembly of *Noctua janthina*, ilNocJanh1.1: metrics. The BlobToolKit snail plot shows N50 metrics and BUSCO gene completeness. The main plot is divided into 1,000 size-ordered bins around the circumference with each bin representing 0.1% of the 539,718,606 bp assembly. The distribution of scaffold lengths is shown in dark grey with the plot radius scaled to the longest scaffold present in the assembly (24,524,560 bp, shown in red). Orange and pale-orange arcs show the N50 and N90 scaffold lengths (18,542,558 and 12,575,359 bp), respectively. The pale grey spiral shows the cumulative scaffold count on a log scale with white scale lines showing successive orders of magnitude. The blue and pale-blue area around the outside of the plot shows the distribution of GC, AT and N percentages in the same bins as the inner plot. A summary of complete, fragmented, duplicated and missing BUSCO genes in the lepidoptera_odb10 set is shown in the top right. An interactive version of this figure is available at https://blobtoolkit.genomehubs.org/view/GCA_963576755.1/dataset/GCA_963576755.1/snail.

**Figure 3.**
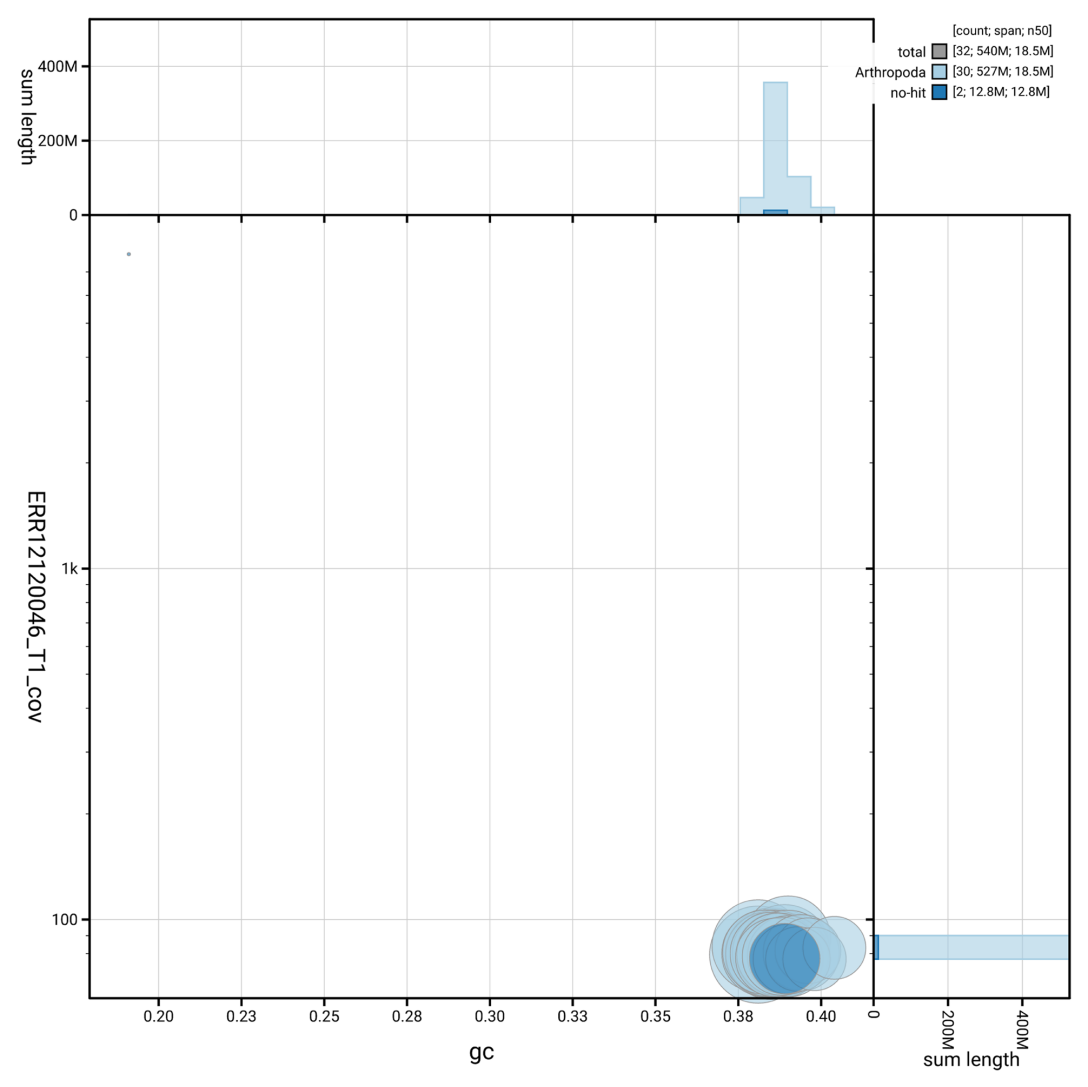
Genome assembly of *Noctua janthina*: Blot plot of base coverage in the raw data against GC proportion for sequences in ilNocJanh1.1. Sequences are coloured by phylum. Circles are sized in proportion to sequence length. Histograms show the distribution of sequence length sum along each axis. An interactive version of this figure is available at https://blobtoolkit.genomehubs.org/view/GCA_963576755.1/dataset/GCA_963576755.1/blob.

**Figure 4.**
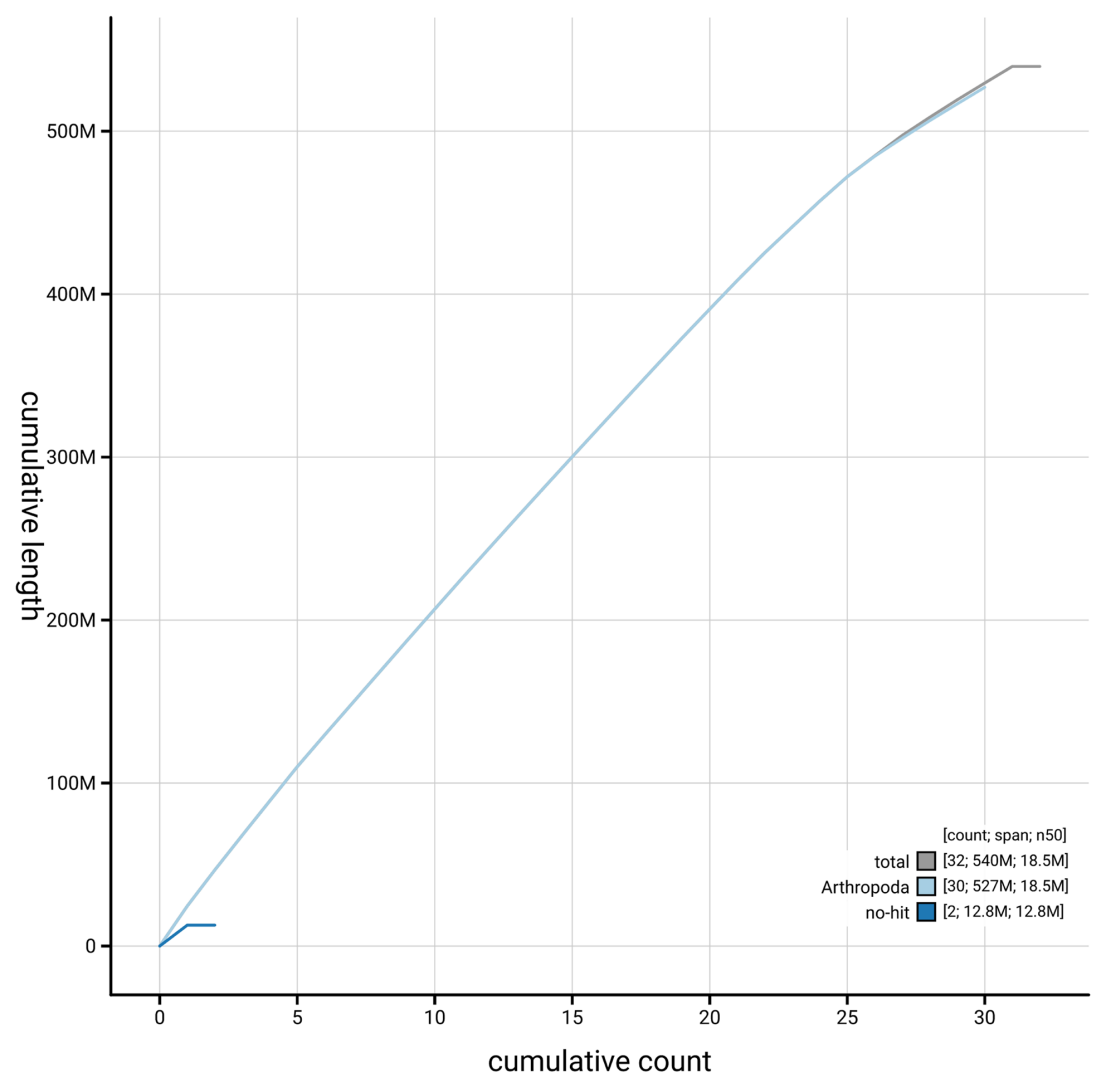
Genome assembly of *Noctua janthina* ilNocJanh1.1: BlobToolKit cumulative sequence plot. The grey line shows cumulative length for all scaffolds. Coloured lines show cumulative lengths of scaffolds assigned to each phylum using the buscogenes taxrule. An interactive version of this figure is available at https://blobtoolkit.genomehubs.org/view/GCA_963576755.1/dataset/GCA_963576755.1/cumulative.

**Figure 5.**
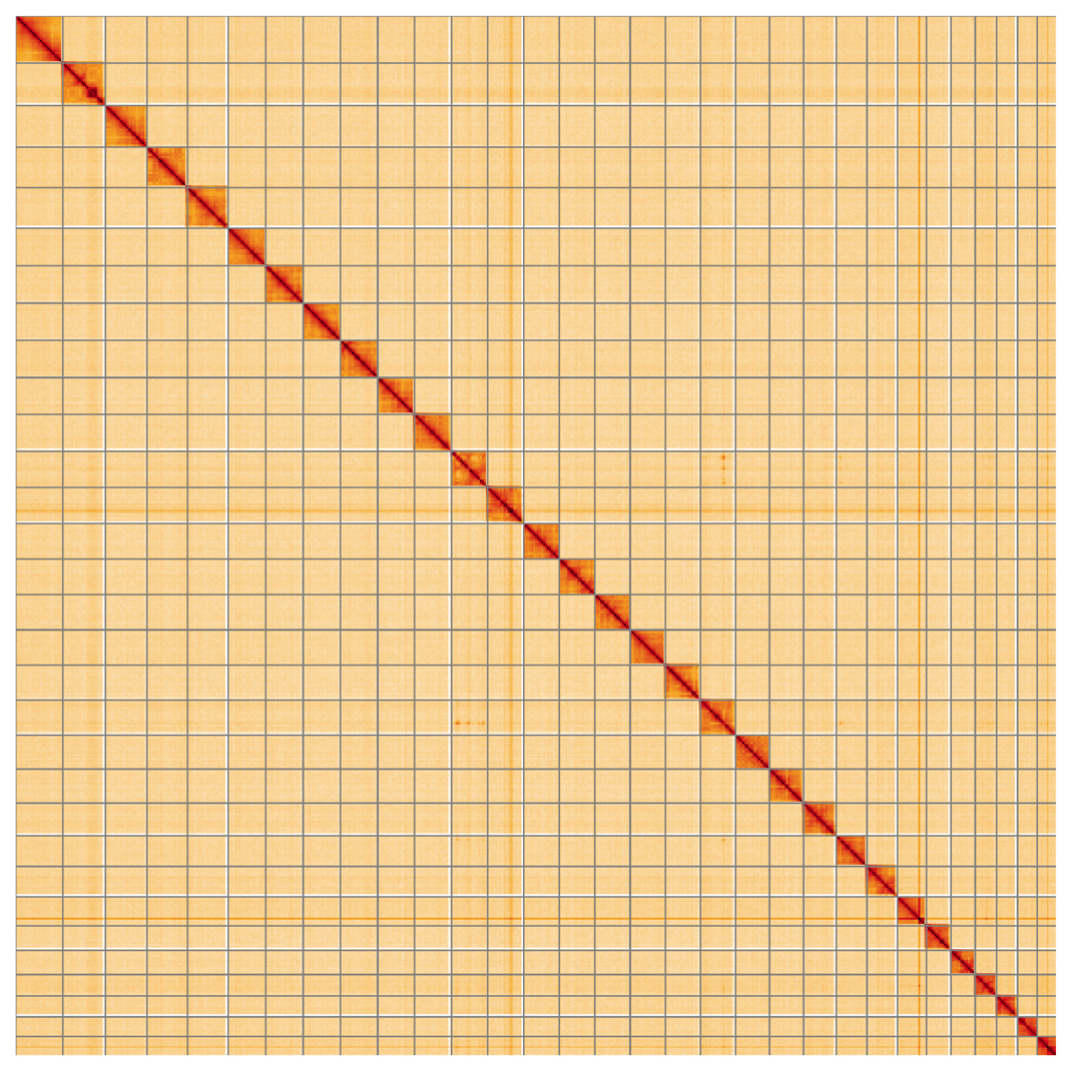
Genome assembly of *Noctua janthina* ilNocJanh1.1: Hi-C contact map of the ilNocJanh1.1 assembly, visualised using HiGlass. Chromosomes are shown in order of size from left to right and top to bottom. An interactive version of this figure may be viewed at https://genome-note-higlass.tol.sanger.ac.uk/l/?d=atPGYJX0RT6rm8L0_J0-zg.

Manual assembly curation corrected 9 missing joins or mis-joins and one haplotypic duplication, reducing the assembly length by 3.83% and the scaffold number by 87.1%. The final assembly has a total length of 539.70 Mb in 31 sequence scaffolds, with 56 gaps, and a scaffold N50 of 18.5 Mb ( Table 2). The snail plot in Figure 2 provides a summary of the assembly statistics, while the distribution of assembly scaffolds on GC proportion and coverage is shown in Figure 3. The cumulative assembly plot in Figure 4 shows curves for subsets of scaffolds assigned to different phyla. Most (99.99%) of the assembly sequence was assigned to 31 chromosomal-level scaffolds, representing 30 autosomes and the Z sex chromosome. Chromosome-scale scaffolds confirmed by the Hi-C data are named in order of size ( Figure 5; Table 3). Z chromosome identified based on synteny with the genome assembly of *Noctua comes* (GCA_963082995.1) ( Boyes *et al.*, 2024). While not fully phased, the assembly deposited is of one haplotype. Contigs corresponding to the second haplotype have also been deposited. The mitochondrial genome was also assembled and can be found as a contig within the multifasta file of the genome submission.

**Table 2.** Genome assembly data for *Noctua janthina*, ilNocJanh1.1.

Genome assembly		
Assembly name	ilNocJanh1.1	
Assembly accession	GCA_963576755.1	
*Accession of alternate haplotype*	*GCA_963576745.1*	
Span (Mb)	539.70	
Number of contigs	88	
Contig N50 length (Mb)	10.1	
Number of scaffolds	31	
Scaffold N50 length (Mb)	18.5	
Longest scaffold (Mb)	24.52	
Assembly metrics *		*Benchmark*
Consensus quality (QV)	69.8	*≥ 50*
*k*-mer completeness	100.0%	*≥ 95%*
BUSCO **	C:98.9%[S:98.3%,D:0.6%], F:0.2%,M:0.9%,n:5,286	*C ≥ 95%*
Percentage of assembly mapped to chromosomes	99.99%	*≥ 95%*
Sex chromosomes	Z	*localised homologous pairs*
Organelles	Mitochondrial genome: 15.36 kb	*complete single alleles*
Genome annotation of assembly GCA_963576755.1 at Ensembl		
Number of protein-coding genes	12,089	
Number of non-coding genes	2,379	
Number of gene transcripts	21,556	

* Assembly metric benchmarks are adapted from column VGP-2020 of “Table 1: Proposed standards and metrics for defining genome assembly quality” from Rhie *et al.* (2021). ** BUSCO scores based on the lepidoptera_odb10 BUSCO set using version 5.4.3. C = complete [S = single copy, D = duplicated], F = fragmented, M = missing, n = number of orthologues in comparison. A full set of BUSCO scores is available at https://blobtoolkit.genomehubs.org/view/Noctua_janthina/dataset/GCA_963576755.1/busco.

**Table 3.** Chromosomal pseudomolecules in the genome assembly of *Noctua janthina*, ilNocJanh1.

INSDC accession	Name	Length (Mb)	GC%
OY755051.1	1	22.1	38.0
OY755052.1	2	21.32	38.5
OY755053.1	3	21.07	39.0
OY755054.1	4	20.99	38.5
OY755055.1	5	19.59	39.0
OY755056.1	6	19.34	38.5
OY755057.1	7	19.33	39.0
OY755058.1	8	19.31	38.5
OY755059.1	9	19.16	38.5
OY755060.1	10	19.04	38.5
OY755061.1	11	18.74	38.5
OY755062.1	12	18.72	39.0
OY755063.1	13	18.54	38.5
OY755064.1	14	18.42	38.5
OY755065.1	15	18.36	38.5
OY755066.1	16	18.26	39.0
OY755067.1	17	18.25	38.5
OY755068.1	18	18.13	39.0
OY755069.1	19	17.65	38.5
OY755070.1	20	17.56	38.5
OY755071.1	21	17.0	39.0
OY755072.1	22	15.9	38.5
OY755073.1	23	15.79	39.0
OY755074.1	24	14.95	39.5
OY755075.1	25	12.79	39.0
OY755076.1	26	12.58	39.0
OY755077.1	27	11.12	39.5
OY755078.1	28	10.72	39.5
OY755079.1	29	10.29	40.0
OY755080.1	30	10.16	40.5
OY755050.1	Z	24.52	38.0
OY755081.1	MT	0.02	19.5

The estimated Quality Value (QV) of the final assembly is 69.8 with *k*-mer completeness of 100.0%, and the assembly has a BUSCO v5.4.3 completeness of 98.9% (single = 98.3%, duplicated = 0.6%), using the lepidoptera_odb10 reference set ( *n* = 5,286).

Metadata for specimens, BOLD barcode results, spectra estimates, sequencing runs, contaminants and pre-curation assembly statistics are given at https://links.tol.sanger.ac.uk/species/987996.

## Genome annotation report

The *Noctua janthina* genome assembly (GCA_963576755.1) was annotated at the European Bioinformatics Institute (EBI) on Ensembl Rapid Release. The resulting annotation includes 21,556 transcribed mRNAs from 12,089 protein-coding and 2,379 non-coding genes ( Table 2; https://rapid.ensembl.org/Noctua_janthina_GCA_963576755.1/Info/Index). The average transcript length is 16,427.94. There are 1.49 coding transcripts per gene and 7.34 exons per transcript.

## Methods

### Sample acquisition and DNA barcoding

An adult male specimen of *Noctua janthina* (specimen ID NHMUK010884576, ToLID ilNocJanh1) was collected from Tonbridge, England, United Kingdom (latitude 51.19, longitude 0.29) on 2022-08-08, using actinic light. The specimen was collected and identified by Gavin Broad (Natural History Museum) and preserved by dry freezing at –80°C.

The initial identification was verified by an additional DNA barcoding process according to the framework developed by Twyford *et al*. (2024). A small sample was dissected from the specimens and stored in ethanol, while the remaining parts were shipped on dry ice to the Wellcome Sanger Institute (WSI). The tissue was lysed, the COI marker region was amplified by PCR, and amplicons were sequenced and compared to the BOLD database, confirming the species identification ( Crowley *et al.*, 2023). Following whole genome sequence generation, the relevant DNA barcode region was also used alongside the initial barcoding data for sample tracking at the WSI ( Twyford *et al.*, 2024). The standard operating procedures for Darwin Tree of Life barcoding have been deposited on protocols.io ( Beasley *et al.*, 2023).

### Nucleic acid extraction

The workflow for high molecular weight (HMW) DNA extraction at the Wellcome Sanger Institute (WSI) Tree of Life Core Laboratory includes a sequence of procedures: sample preparation and homogenisation, DNA extraction, fragmentation and purification. Detailed protocols are available on protocols.io ( Denton *et al.*, 2023b). The ilNocJanh1 sample was prepared for DNA extraction by weighing and dissecting it on dry ice ( Jay *et al.*, 2023) and tissue from the whole organism was homogenised using a PowerMasher II tissue disruptor ( Denton *et al.*, 2023a).

HMW DNA was extracted in the WSI Scientific Operations core using the Automated MagAttract v2 protocol ( Oatley *et al.*, 2023). The DNA was sheared into an average fragment size of 12–20 kb in a Megaruptor 3 system ( Bates *et al.*, 2023). Sheared DNA was purified by solid-phase reversible immobilisation, using AMPure PB beads to eliminate shorter fragments and concentrate the DNA ( Strickland *et al.*, 2023). The concentration of the sheared and purified DNA was assessed using a Nanodrop spectrophotometer and Qubit Fluorometer using the Qubit dsDNA High Sensitivity Assay kit. Fragment size distribution was evaluated by running the sample on the FemtoPulse system.

### Hi-C preparation

Hi-C data were generated from tissue derived from the whole organism of ilNocJanh1, using the Arima-HiC v2 kit. In brief, frozen tissue (stored at –80°C) was fixed, and the DNA crosslinked using a TC buffer with 22% formaldehyde concentration. After crosslinking the tissue was homogenised using the Diagnocine Power Masher-II and BioMasher-II tubes and pestles. Following the kit manufacturer's instructions, crosslinked DNA was digested using a restriction enzyme master mix. The 5’-overhangs were then filled in and labelled with biotinylated nucleotides and proximally ligated. An overnight incubation was carried out for enzymes to digest remaining proteins and for crosslinks to reverse. A clean up is performed with SPRIselect beads prior to library preparation.

### Library preparation and sequencing

Pacific Biosciences HiFi circular consensus DNA sequencing libraries were constructed according to the manufacturers’ instructions. DNA sequencing was performed by the Scientific Operations core at the WSI on a Pacific Biosciences Revio instrument.

For Hi-C library preparation, DNA was fragmented to a size of 400 to 600 bp using a Covaris E220 sonicator. The DNA was then enriched, barcoded, and amplified using the NEBNext Ultra II DNA Library Prep Kit following manufacturers’ instructions. The Hi-C sequencing was performed using paired-end sequencing with a read length of 150 bp on an Illumina NovaSeq 6000.

### Genome assembly, curation and evaluation

***Assembly***

The HiFi reads were first assembled using Hifiasm ( Cheng *et al.*, 2021) with the --primary option. Haplotypic duplications were identified and removed using purge_dups ( Guan *et al.*, 2020). The Hi-C reads were mapped to the primary contigs using bwa-mem2 ( Vasimuddin *et al.*, 2019). The contigs were further scaffolded using the provided Hi-C data ( Rao *et al.*, 2014) in YaHS ( Zhou *et al.*, 2023) using the --break option. The scaffolded assemblies were evaluated using Gfastats ( Formenti *et al.*, 2022), BUSCO ( Manni *et al.*, 2021) and MERQURY.FK ( Rhie *et al.*, 2020).

The mitochondrial genome was assembled using MitoHiFi ( Uliano-Silva *et al.*, 2023), which runs MitoFinder ( Allio *et al.*, 2020) and uses these annotations to select the final mitochondrial contig and to ensure the general quality of the sequence.

***Assembly curation***

The assembly was decontaminated using the Assembly Screen for Cobionts and Contaminants (ASCC) pipeline (article in preparation). Flat files and maps used in curation were generated in TreeVal ( Pointon *et al.*, 2023). Manual curation was primarily conducted using PretextView ( Harry, 2022), with additional insights provided by JBrowse2 ( Diesh *et al.*, 2023) and HiGlass ( Kerpedjiev *et al.*, 2018). Scaffolds were visually inspected and corrected as described by Howe *et al*. (2021). Any identified contamination, missed joins, and mis-joins were corrected, and duplicate sequences were tagged and removed. The sex chromosome was identified by synteny analysis. The curation process is documented at https://gitlab.com/wtsi-grit/rapid-curation (article in preparation).

***Evaluation of the final assembly***

The final assembly was post-processed and evaluated using the three Nextflow ( Di Tommaso *et al.*, 2017) DSL2 pipelines: sanger-tol/readmapping ( Surana *et al.*, 2023a), sanger-tol/genomenote ( Surana *et al.*, 2023b), and sanger-tol/blobtoolkit ( Muffato *et al.*, 2024). The readmapping pipeline aligns the Hi-C reads using bwa-mem2 ( Vasimuddin *et al.*, 2019) and combines the alignment files with SAMtools ( Danecek *et al.*, 2021). The genomenote pipeline converts the Hi-C alignments into a contact map using BEDTools ( Quinlan & Hall, 2010) and the Cooler tool suite ( Abdennur & Mirny, 2020). The contact map is visualised in HiGlass ( Kerpedjiev *et al.*, 2018). This pipeline also generates assembly statistics using the NCBI datasets report ( Sayers *et al.*, 2024), computes *k*-mer completeness and QV consensus quality values with FastK and MERQURY.FK, and runs BUSCO ( Manni *et al.*, 2021) to assess completeness.

The blobtoolkit pipeline is a Nextflow port of the previous Snakemake Blobtoolkit pipeline ( Challis *et al.*, 2020). It aligns the PacBio reads in SAMtools and minimap2 ( Li, 2018) and generates coverage tracks for regions of fixed size. In parallel, it queries the GoaT database ( Challis *et al.*, 2023) to identify all matching BUSCO lineages to run BUSCO ( Manni *et al.*, 2021). For the three domain-level BUSCO lineages, the pipeline aligns the BUSCO genes to the UniProt Reference Proteomes database ( Bateman *et al.*, 2023) with DIAMOND ( Buchfink *et al.*, 2021) blastp. The genome is also split into chunks according to the density of the BUSCO genes from the closest taxonomic lineage, and each chunk is aligned to the UniProt Reference Proteomes database with DIAMOND blastx. Genome sequences without a hit are chunked with seqtk and aligned to the NT database with blastn ( Altschul *et al.*, 1990). The blobtools suite combines all these outputs into a blobdir for visualisation.

The genome assembly and evaluation pipelines were developed using nf-core tooling ( Ewels *et al.*, 2020) and MultiQC ( Ewels *et al.*, 2016), relying on the Conda package manager, the Bioconda initiative ( Grüning *et al.*, 2018), the Biocontainers infrastructure ( da Veiga Leprevost *et al.*, 2017), as well as the Docker ( Merkel, 2014) and Singularity ( Kurtzer *et al.*, 2017) containerisation solutions.

Table 4 contains a list of relevant software tool versions and sources.

**Table 4.** Software tools: versions and sources.

Software tool	Version	Source
BEDTools	2.30.0	https://github.com/arq5x/bedtools2
BLAST	2.14.0	ftp://ftp.ncbi.nlm.nih.gov/blast/executables/blast+/
BlobToolKit	4.3.7	https://github.com/blobtoolkit/blobtoolkit
BUSCO	5.4.3 and 5.5.0	https://gitlab.com/ezlab/busco
bwa-mem2	2.2.1	https://github.com/bwa-mem2/bwa-mem2
Cooler	0.8.11	https://github.com/open2c/cooler
DIAMOND	2.1.8	https://github.com/bbuchfink/diamond
fasta_windows	0.2.4	https://github.com/tolkit/fasta_windows
FastK	427104ea91c78c3b8b8b49f1a7d6bbeaa869ba1c	https://github.com/thegenemyers/FASTK
Gfastats	1.3.6	https://github.com/vgl-hub/gfastats
GoaT CLI	0.2.5	https://github.com/genomehubs/goat-cli
Hifiasm	0.19.8-r587	https://github.com/chhylp123/hifiasm
HiGlass	44086069ee7d4d3f6f3f0012569789ec138f42b84aa44357826c0b6753eb28de	https://github.com/higlass/higlass
Merqury.FK	d00d98157618f4e8d1a9190026b19b471055b22e	https://github.com/thegenemyers/MERQURY.FK
MitoHiFi	3	https://github.com/marcelauliano/MitoHiFi
MultiQC	1.14, 1.17, and 1.18	https://github.com/MultiQC/MultiQC
NCBI Datasets	15.12.0	https://github.com/ncbi/datasets
Nextflow	23.04.0-5857	https://github.com/nextflow-io/nextflow
PretextView	0.2	https://github.com/sanger-tol/PretextView
purge_dups	1.2.5	https://github.com/dfguan/purge_dups
samtools	1.16.1, 1.17, and 1.18	https://github.com/samtools/samtools
sanger-tol/ascc	-	https://github.com/sanger-tol/ascc
sanger-tol/genomenote	1.1.1	https://github.com/sanger-tol/genomenote
sanger-tol/readmapping	1.2.1	https://github.com/sanger-tol/readmapping
Seqtk	1.3	https://github.com/lh3/seqtk
Singularity	3.9.0	https://github.com/sylabs/singularity
TreeVal	1.0.0	https://github.com/sanger-tol/treeval
YaHS	1.2a.2	https://github.com/c-zhou/yahs

### Genome annotation

The Ensembl Genebuild annotation system ( Aken *et al.*, 2016) was used to generate annotation for the *Noctua janthina* assembly (GCA_963576755.1) in Ensembl Rapid Release at the EBI. Annotation was created primarily through alignment of transcriptomic data to the genome, with gap filling via protein-to-genome alignments of a select set of proteins from UniProt ( UniProt Consortium, 2019).

### Wellcome Sanger Institute – Legal and Governance

The materials that have contributed to this genome note have been supplied by a Darwin Tree of Life Partner. The submission of materials by a Darwin Tree of Life Partner is subject to the **‘Darwin Tree of Life Project Sampling Code of Practice’**, which can be found in full on the Darwin Tree of Life website here. By agreeing with and signing up to the Sampling Code of Practice, the Darwin Tree of Life Partner agrees they will meet the legal and ethical requirements and standards set out within this document in respect of all samples acquired for, and supplied to, the Darwin Tree of Life Project. 

Further, the Wellcome Sanger Institute employs a process whereby due diligence is carried out proportionate to the nature of the materials themselves, and the circumstances under which they have been/are to be collected and provided for use. The purpose of this is to address and mitigate any potential legal and/or ethical implications of receipt and use of the materials as part of the research project, and to ensure that in doing so we align with best practice wherever possible. The overarching areas of consideration are:

• Ethical review of provenance and sourcing of the material

• Legality of collection, transfer and use (national and international)

Each transfer of samples is further undertaken according to a Research Collaboration Agreement or Material Transfer Agreement entered into by the Darwin Tree of Life Partner, Genome Research Limited (operating as the Wellcome Sanger Institute), and in some circumstances other Darwin Tree of Life collaborators.

## Data Availability

European Nucleotide Archive: *Noctua janthina.* Accession number PRJEB67419; https://identifiers.org/ena.embl/PRJEB67419 ( Wellcome Sanger Institute, 2023). The genome sequence is released openly for reuse. The *Noctua janthina* genome sequencing initiative is part of the Darwin Tree of Life (DToL) project. All raw sequence data and the assembly have been deposited in INSDC databases. Raw data and assembly accession identifiers are reported in Table 1 and Table 2.

## Author information

Members of the Natural History Museum Genome Acquisition Lab are listed here: https://doi.org/10.5281/zenodo.12159242.

Members of the Wellcome Sanger Institute Tree of Life Management, Samples and Laboratory team are listed here: https://doi.org/10.5281/zenodo.12162482.

Members of Wellcome Sanger Institute Scientific Operations: Sequencing Operations are listed here: https://doi.org/10.5281/zenodo.12165051.

Members of the Wellcome Sanger Institute Tree of Life Core Informatics team are listed here: https://doi.org/10.5281/zenodo.12160324.

Members of the Tree of Life Core Informatics collective are listed here: https://doi.org/10.5281/zenodo.12205391.

Members of the Darwin Tree of Life Consortium are listed here: https://doi.org/10.5281/zenodo.4783558.
