# Supplementary material for: The genome sequence of Langmaid’s Yellow Underwing moth, Noctua janthina (Denis & Schiffermüller) 1775
Source: Wellcome Open Res. 2024 Oct 15;9:592. [Version 1] doi: 10.12688/wellcomeopenres.23192.1 (PMC11569387; doi:10.12688/wellcomeopenres.23192.1)
Supplement: Peer review history

# Reviewer report: Reviewer response for version 1

**DOI:** 10.21956/wellcomeopenres.25542.r109935

**Published:** 2024-11-26

**Authors:** Annabel Whibley

**Affiliations:** 1 Grapevine Improvement, Bragato Research Institute, Lincoln, New Zealand; 2 The University of Auckland, Auckland, Auckland, New Zealand

In this Data Note, Broad and colleagues report the genome assembly and gene annotation of Langmaid’s Yellow Underwing moth, *Noctua janthina*.

 The background discussion of natural history and taxonomy is engaging and provides useful context to the study species.

 The genome assembly appears to be of extremely high quality, with impressive completeness, contiguity and accuracy statistics. The HiC contact map and Blobplot are both very clean visualizations and attest to the assembly quality. Annotation has been performed without RNA evidence via the Ensembl pipeline.

 Methods are  well-described and appropriate, sample and analysis metadata is carefully recorded and the links to sequence and assembly accessions are active. I welcome the addition of the DNA barcode verification step into the assembly pipeline, this seems a sensible level of quality checking to implement, especially in taxonomically challenging lineages such as this.

Are sufficient details of methods and materials provided to allow replication by others?

Yes

Is the rationale for creating the dataset(s) clearly described?

Yes

Are the datasets clearly presented in a useable and accessible format?

Yes

Are the protocols appropriate and is the work technically sound?

Yes

Reviewer Expertise:

Genomics, Bioinformatics

I confirm that I have read this submission and believe that I have an appropriate level of expertise to confirm that it is of an acceptable scientific standard.

# Reviewer report: Reviewer response for version 1

**DOI:** 10.21956/wellcomeopenres.25542.r108535

**Published:** 2024-11-16

**Authors:** Pritha Dey

**Affiliations:** 1 National Centre for Biological Sciences, Kodigehalli, India

The genome assembly of the male *Noctua janthina* (Langmaid’s Yellow Underwing), a species of moth in the family *Noctuidae*, presents several key findings. The genome is 539.70 mega bases in total length, with 99.99% of the sequence scaffolded into 31 chromosomal pseudomolecules, including the Z sex chromosome. This suggests a high-quality, well-assembled genome. The mitochondrial genome has been successfully assembled, with a length of 15.36 kilobases, providing insights into the mitochondrial DNA structure of this species. A comprehensive gene annotation identified 12,089 protein-coding genes within the genome. This data is essential for understanding the functional biology of *N. janthina* and contributes to the broader understanding of Lepidopteran genetics.

 The genome sequence enriches the genetic understanding of the *Noctuidae* family and *Lepidoptera* as a whole, offering valuable data for comparative genomics within this order. The methods are very clear, detailed and easily replicable.

 ​​​​​​The species description is particularly useful. Understanding the genome of *Noctua janthina* could aid in the development of pest control strategies, particularly because members of the *Noctuidae* family are known agricultural pests.

Are sufficient details of methods and materials provided to allow replication by others?

Yes

Is the rationale for creating the dataset(s) clearly described?

Yes

Are the datasets clearly presented in a useable and accessible format?

Yes

Are the protocols appropriate and is the work technically sound?

Yes

Reviewer Expertise:

DNA barcoding, evolutionary ecology, morphological traits

I confirm that I have read this submission and believe that I have an appropriate level of expertise to confirm that it is of an acceptable scientific standard.

# Reviewer report: Reviewer response for version 1

**DOI:** 10.21956/wellcomeopenres.25542.r107135

**Published:** 2024-11-01

**Authors:** Axel Künstner

**Affiliations:** 1 University of Lübeck, Lübeck, Germany

The manuscript describes the genome assembly of Langmaid’s Yellow Underwing moth ( *Noctua janthina*). To assemble the genome, a single male individual was used, and the assembly comprises a total length of 539.70 MB. As males are the homogametic sex in *Noctua janthina*, the assembly lacks the W sex chromosome. The mitochondrial genome is provided as well with the nuclear genome assembly.

 The methods used are sound, software resources used are well described, and the data is available via the European Nucleotide Archive (Accession number PRJEB67419).

Are sufficient details of methods and materials provided to allow replication by others?

Yes

Is the rationale for creating the dataset(s) clearly described?

Yes

Are the datasets clearly presented in a useable and accessible format?

Yes

Are the protocols appropriate and is the work technically sound?

Yes

Reviewer Expertise:

Cancer genomics, Microbiome, Bioinformatics, Computational Biology

I confirm that I have read this submission and believe that I have an appropriate level of expertise to confirm that it is of an acceptable scientific standard.
